# Assessing the impact of governance and health expenditures on carbon emissions in China: Role of environmental regulation

**DOI:** 10.3389/fpubh.2022.949729

**Published:** 2022-09-06

**Authors:** Yue Wang, Mengjie Liu, Shan Wang, Xiuping Cui, Lei Hao, HaSiBa Gen

**Affiliations:** ^1^School of Resources and Environmental Economics, Inner Mongolia University of Finance and Economics, Hohhot, China; ^2^Resource Utilization and Environmental Protection Coordinated Development Academician Expert Workstation in the North of China, Inner Mongolia University of Finance and Economics, Inner Mongolia, Hohhot, China; ^3^School of Economics and Management, Beijing Forestry University, Beijing, China

**Keywords:** health expenditures, governance, income, rule of law, China

## Abstract

The current study tries to summarize the leading factors and their behavior toward the environmental quality. Therefore, this study investigates the environment-development linkage in China's economy covering the period of 1984–2018. In order to investigate the proposed objectives, the current study uses the dynamic ordinary least square, fully modified ordinary least square and canonical co-integration regression with granger causality test. The results reveal that there exists an inverted U-shaped relationship in China's economy. Likewise, urbanization positively contributes to environmental deterioration. Furthermore, the health expenditures also cause to deteriorate the environmental quality. In contrast, there exists the negative association between good governance and carbon emissions, while the rule of law in China's economy does not secure the environmental quality. Therefore, environmental regulation policies need to be revised in order to achieve a sustainable environment. As a result, we recommend that China continue to expand its economy and invest in health care and environmental initiatives.

## Introduction

Humanity is facing a worldwide environmental challenge in the form of global warming (1). China, both the largest developing country and the country that emits the most carbon into the atmosphere, has pledged to take various significant steps to decrease the effects of climate change. Specifically, during the climate change conference in Paris in November 2015, China proclaimed that she would reach her peak carbon discharge level around the year 2030 and strive to achieve this peak earlier rather than later (2). If the path chosen to reach that goal is appropriate, achieving this goal will not negatively impact China's fiscal or social development. This is because China's goal is to become a developed nation by 2020 (3). Instead, achieving the goal will encourage a shift in China's pattern of economic development toward green growth and low carbon emissions (4). That is why China is considered the world's largest emitter of carbon dioxide (5). Reducing carbon emissions at the provincial level is crucial for China to achieve its goal in terms of policy, scenario, and city. Through private voluntary actions, non-governmental organizations, and top-down management, China has been an active participant in worldwide climate governance efforts to combat climate change (6). There have been clear impacts on China's overall CO2 discharges as a result of the dramatic changes that occurred in the structure of the Chinese economy in newly emerging sectors.

Since the 1970's, there has been a significant amount of interest from researchers and practitioners in the field of research regarding the associations between environmental excellence and economic development. A steady-state economy is advocated for in the book “Limits to Growth” to prevent environmental destruction in the upcoming period. Over the last decade, the economies of many developing nations have experienced rapid expansion (7). Since it first implemented its policy of reform and opening to the outside world in 1978, China, one of the largest developing countries, has gone through a period of rapid change. China's GDP grew at the rate of 9.7 percent per year over the course of the last 20 years, propelling the country into the top 10 list of the world's largest economies (8). However, this rapid expansion has resulted in the depletion of significant amounts of resources and an increase in the amount of pollution produced. The emission of excessive amounts of greenhouse gases has accelerated the degradation of the environment and the progression of climate change, which constitutes a risk to the wellbeing of humans and the stability of the global community. The negative effects of carbon secretions on health can generally be broken down into two main categories. First, breathing in high concentrations of carbon dioxide can cause direct damage to a person's respiratory system, leading to shortness of breath, headaches, and even delirium (9). The second way is more covert and is associated with the changing climate. A steady increase in carbon emissions will, on the one hand, bring about global warming, bring about changes in the amount of precipitation and the pattern of precipitation, and bring about increase the frequency of extreme weather. In 2018, high temperatures and heatwaves affected 157 million people worldwide (10). Experiments in the medical field suggest that exposure to high temperatures for extended periods raises the risk of developing respiratory and cardiovascular diseases (9). The unpredictable results of extreme weather, such as drought, floods, and storms, can also be brought about by these natural phenomena. On the other side, global warming will worsen air pollution and speed up the spread of diseases, which will indirectly threaten human health. According to projections made by the WHO, climate change will be directly responsible for approximately 250,000 deaths per year between the years 2030 and 2050 (11). As a result, institutions can improve their performance to secure the environment better and its potentially harmful impact on human beings.

Institutional factors in environmental pollution have become more prominent in recent years. Institutions have the potential to either directly or indirectly affect the quality of the environment through the implementation of policies and regulations. The body of existing research uses many different stand-ins to evaluate the quality of institutions. As far as governance structures go, corruption and the rule of law are important indicators. These pillars of governance are just two of many that make up a governance structure (12, 13). When it comes to the formulation and execution of environmental policies, having an institutional system that is functional and objective is of the utmost importance. A stable and strong government can formulate an efficient environmental strategy with a culture free of corruption; a country with a strict rule of law can then impose the strategy. On the other hand, companies violate pollution control protocols to achieve maximum revenue if there are flaws in the system and weak institutions (14). In addition, strong institutions cut pollution on the home front and have the potential to spread the effect to countries adjacent to the United States through a phenomenon known as the spatial institutional spillover effect (15). During the process of economic expansion, the contribution of institutions that are both objective and efficient to the reduction of environmental pollution is invaluable (16, 17). On the other hand, weak institutes, which are regarded as the primary cause of the low profits, are the primary impediments to the formulation and regulation of environmental strategies, the adoption of environmentally friendly technologies, and the development of an advanced energy structure (18). Because of this, having strong institutions in a country is necessary and extremely important in order to control the amount of environmental pollution. There is a close connection between monetary growth and the excellence of the surrounding environment. In the past 10 years, a significant amount of research has been conducted in China on the country's severe environmental deterioration and the various approaches that have been taken to address it. But only a small number of real-world empirical studies have used environmental Kuznets curve (ENVIRONMENT KUZNETS CURVE) analysis to examine the relationship between rising economic activity and various pollution indicators in China. Decomposition analysis of ENVIRONMENT KUZNETS CURVE is used to investigate the causal relationship between economic development and the main pollutants. With the help of a case study from China, the author hopes to shed some light on the difficulties of balancing economic growth with environmental protection.

The level of income is shown to have a significant impact on the relationship between institutions and environmental pollution, according to evidence that is both empirical and theoretical. According to Yu and Wang (6), countries with higher quality institutions are better able to reduce environmental pollution effectively. This study aims to study the link between income level and institutional factors. In terms of originality, this would be the first study to include three separate institutional variables and examine the impact that each of those variables has on carbon emissions in China.

The assessments of the impacts of emissions in China are still lacking, although the country's contributions to overall emissions are growing, and the associated health risks are rising. There is a lack of accurate estimations of emissions in China, which is one of the reasons for the limited understanding of the impacts of emissions. Previous studies have taken data from global inventories and extracted China's emissions from there (15, 19). In more recent times, emissions calculations have been based on economic factors and the amount of energy consumed (20). Consequently, it was estimated that emissions from solid fuels would have a significant impact (21). The absence of a reliable method for quantifying the health impact specifically caused by emissions is another factor contributing to the inconsistency of previous research. Previous studies assumed no difference in the risk associated with an increase in concentration when calculating the number of premature deaths caused by exposure to various PM components (22, 23). It is necessary, as a result, to develop a comprehensive emission inventory in China in order to conduct an analysis of the impact on health expenditures caused by emissions. Likewise, a case study of China provinces tried to investigate the role of governance in low carbon emissions. They demonstrated that local government relay on strong financial support. Likewise, the industrial transformation strongly based on green governance. The impact of green governance pear effect decreases due to rising geographical distance. Moreover, this study tries to focus on different regions of China i.e., eastern, western, and central regions and found the diminishing impact of green governance peer effects. The outcomes of the study are very ambiguous and it is need of time to select some clear indicators to minimize the environmental damages. Therefore, this study has made an effort to clear such ambiguous behavior of variables and straightforward policy implications for sustainable environment. Therefore, this study tries to investigate the important role of governance and rule of law to check out their response to carbon emissions, because rule of law and good governance are being considered the extremely supportive instruments to minimize the negative externalities as a result of economic development. Moreover, without any base theory the empirical study may produce misleading outcomes; so, the current study also tries to investigate the role of governance and rule of law under the prism of Environment Kuznets Curve hypothesis. This is a new contribution of the current study to existing literature that the main target of each economy (economic development) how behaves with environment level. Similarly, the urbanization and health expenditures are also directly linked with environmental degradation. Therefore, this is another significant contribution by this study to existing literature and tries to explain the behavior of urbanization and health expenditure to environmental deterioration. Finally, in order to investigate such interesting contributions to existing literature, this study uses a series of econometric techniques for the robust outcomes in which the FMOLS, DOLS, and CCR regressions. In addition, the Granger causality test was utilized in this investigation to investigate the causal association between the variables that were of concern.

Similarly, this study organized as follows, section 2 describes the literature on previous studies, section 3 presents the data and methods, section 4 shows the results and discussion and section 5 describes the conclusion and policy recommendations.

## Literature review

### The health-environment nexus

It is critical to have a solid understanding of this health effect because it may provide extra information that can be used to advance the consistency and effectiveness of environmental policies and medical care. The literature contains a large number of studies (24) that investigate the correlation between people's health and the quality of their surrounding environment; though, the majority of the studies that have been conducted to date have concentrated on the environmental risks posed by air pollutants (such as PM2.5 and sulfur dioxide, amongst others) (25, 26). Emissions of carbon, which make up a significant portion of greenhouse gases, are likely to be the source of health issues. Despite this, there hasn't been a lot of research done on the link between the health of locals and carbon emissions, and most of it has focused on medical topics. Emissions of carbon have a direct and negative impact on the ability of humans to function physically. In addition, exposure to high concentrations of CO2 can result in symptoms such as headache, dizziness, fatigue, weakness, and distraction (27, 28). Ehigiamusoe et al. (25) found that employees working in low-carbon offices had higher cognitive function scores. They also found that even a little quantity of carbon dioxide could impair a person's decision-making ability. Additionally, qualifying as a “villain” of global warming and climate change is carbon dioxide. People who live in environments where the temperature remains consistently high have an increased risk of developing diseases that affect the nervous, cardiovascular, digestive, and respiratory systems (29, 30). Both food production and food security are in danger as a result of global warming. It is possible that higher temperatures, which create a more favorable environment for growth and reproduction, will lead to an increase in the number of infectious pathogens as well as insect vectors, which will hasten the spread of infectious diseases like malaria and dengue fever (31, 32). However, identifying this process is very challenging. It is only possible to get a rough estimate based on how frequently and for how long the atmospheric homeostasis occurs. In addition, there is the possibility that climate change will bring about a multitude of risks in the form of climate migration, increased poverty levels, violent conflicts, and mental illness (5, 33). As a result, emissions of carbon dioxide are not only a problem for the environment but also for the health and happiness of people. It's possible that lowering carbon emissions will lead to significant improvements in occupants' health (34).

Research on the link between carbon emissions and locals' health has also been conducted in atmospheric science and geography science, whereas economics research has been conducted less frequently. According to the findings of an investigation that (35) carried out using a global, regional model, CO2 significantly worsens the levels of ozone and other particulate matter in areas already polluted. It is estimated that carbon emissions are responsible for ~21,600 deaths per year across the globe. Hu et al. (10) utilized global atmospheric model simulations and concluded that a reduction in global greenhouse gas emissions has the potential to lower the average death rate effectively. Panel data from (36) showed that rising healthcare costs are linked to carbon emissions, harming residents' health. They also found that carbon emissions are harmful to the health of residents (37) utilized quasi-natural experiments and demonstrated that the continuous discharge of greenhouse gases is to blame for the rising infant mortality rate that has been observed in the United States. Researchers in Malaysia (38), Pakistan (39), and other countries in Sub-Saharan Africa found a link between carbon emissions and medical spending that was statistically significant (40, 41). Furthermore, researchers frequently use the autoregressive distributed lagged (ARDL) model (42). On the other hand, the research presented above merely incorporated a nation or a region into the framework of the analysis; more specifically, it did not consider the diversity of regional economic development. According to earlier empirical research, it is common knowledge that an increase in the rate of global warming caused by a higher concentration of CO_2_ in the atmosphere poses a threat to both humans and ecologies. However, the methods from atmospheric science, geography, and medicine were typically applied in this literature; only a few studies considered an econometric panel analysis. More importantly, not even a single other study has found anything remotely similar to what this one has.

### The environmental regulations-environment nexus

Numerous researchers have investigated the connection between CO_2_ discharges and various other factors in light of the growing severity of the effects of global warming. The majority of researchers concentrated their attention on determining the connection between environmental regulations and carbon emissions (43, 44). Sinn first put forward the idea of the “green paradox” in 1998. According to this theory, environmental regulatory policies designed to reduce carbon emissions will result in a significant increase in carbon emissions. For instance, Hermundsdottir and Aspelund (45) discovered that the green development plans implemented in South Korea resulted in significant emissions of greenhouse gases. In addition to this, It was found by Nasir et al. (46) that green policies can lead countries without strict environmental regulations to increase their use of fossil fuels, thereby increasing global CO_2_ emissions. Salari et al. (47) was found to be the case when countries increased their use of fossil fuels in response to the implementation of environmental policies (such as subsidies or taxes). On the other hand, a number of researchers have discovered that environmental regulation can successfully reduce CO_2_ emissions (38, 48). Jinru et al. (49), the most recent study before the Chinese one, suggested that environmental regulation could negatively affect the environment for economic regions that are already environmentally depressed. According to Anser et al. (50), environmental regulation has a direct effect on carbon emissions, and the effect has shifted from the “green paradox” to “the reverse of pollution reduction.” This suggests that the effect has a negative correlation with pollution reduction. According to Sulich and Sołoducho-Pelc (51), regional differences can lead to different effects of environmental regulation, and increase in the intensity of environmental regulation will lead to an increase in carbon emissions in some regions. This was another point brought up in their study. As a result, a number of researchers concluded that environmental regulations and carbon emissions have a significant relationship that resembles an inverted “U” curve. That is to say, the effect known as the “green paradox” appears before the inflection point, whereas the effect known as the “reverse of emission reduction” appears after the inflection point (52, 53). The majority of these studies disregarded the possibility that one regional environmental regulation might affect another regional environmental regulation, which in turn might affect carbon emissions. The majority of the currently available studies focus on the influence that environmental governance has on its interaction with environmental regulation. Those cities that prioritize protection of the environment are more vulnerable to the effects of less stringent environmental regulations in their neighboring cities, according to the research results of (54). It has been suggested that regional competition could significantly decrease haze control's effectiveness Irani and Kilic (55). While this may be the case (56) found that regulatory governance has a major strategic interaction between regions that positively affects environmental governance. A more effective approach to environmental management may result from this interaction.

In conclusion, a significant amount of research has been carried out by a number of academics on the interactive behaviors of environmental regulation; however, there are not many studies that discuss the impact that environmental regulatory connections have on carbon emissions. In most of the research that has been done, the influence of environmental regulation on carbon emissions has been examined in the context of individual local governments. This approach, however, ignores other significant factors that will also affect the level of emissions. Will it affect CO_2_ emissions, and if so, what is the mechanism by which it will have that effect? The existing body of research does not provide a satisfactory answer to these questions. In order to investigate the effect that environmental regulatory interactions have on CO_2_ emissions, the data for the time series are taken from 1984 up until 2018.

### The governance-environment nexus

Not only do economic considerations play a role in the successful improvement of environment quality (EQ), but policymakers and other decision-makers in government also provide important parameters. This is because environmental governance falls under the purview of the government. According to Brunner, The “innovation compensation effect,” as proposed by Gupta et al. (57), could lead to an increase in environmentally friendly technology manufacturing in other countries as a result of government regulation of the environment. This would have a positive effect on the environment as a whole. Central government officials are in charge of environmental protection in China's environmental governance system, while local governments play a key role in achieving environmental protection goals at the local level (58, 59). The Environmental Protection Law makes it abundantly clear that the responsibility for the quality of the environment in each of the respective administrative regions lies with the respective local governments. In the end, the economy of the area and its quality of life are impacted by factors such as the behavior, power, and governance experience of officials in the local government (60). Based on information gathered from the various industries and provinces in China, concluded that governmental spending on environmental protection has a substantial impact on the reduction of pollution.

When working to improve environmental governance, it is important not to discount public participation's influence. Participation from the public is essential for maintaining flexibility when resolving environmental issues (ref). This helps decision-makers increase their knowledge and competence, which prompts them to distinguish public concerns and demands (61). According to Wang et al. (62), the public should have the right to 'vote with their feet and select public services that are in accordance with the preferences that the public has (63) believed that ENGOs play an important role in indorsing EQ and changing individual behaviors and living styles. They considered ENGOs to be a part of public participation. In a series of case studies conducted in Indonesia (64), discovered that the step taken by residents and environmental non-governmental organizations (ENGOs) had the potential to improve the quality of the environmental. This paper and Dudek and Spiewak (65) both analyze the impact on regional environmental quality from government governance and public participation. However, Irani and Kilic (54) only focused on characterizing government environmental governance from the implementation of local government environmental responsibility goals, and they neglected the combined effect that public participation has on government governance. This paper analyses the impact on regional environmental quality from the perspectives of public participation and government governance. In conclusion (66) held that public demands help improve the effectiveness of environmental governance while also contributing to a limited decrease in the amount of energy consumed.

The perception of how well a society adheres to the law, particularly in policy and regulation enforcement, is one of the factors that contribute to the rule of law (67, 68). The rule of law is an important component of any institution, and it is instrumental in putting environmental policies into effect. A robust rule of law helps ensure that policies are implemented as intended and compels businesses to adhere to environmental policy guidelines. A robust rule of law contributes to reducing carbon emissions by ensuring that businesses adhere to the established guidelines for pollution control (69). The research conducted by Alam et al. (70) discovered that having a robust rule of law contributes to reducing carbon emissions and stimulating economic growth. However, Iqbal et al. (71) concluded that democracies and the rule of law both contribute to an increase in CO_2_ emissions, whereas political stability and the control of corruption both contribute to a decrease. Khurana et al. (72) provides further evidence that the rule of law has a harmful effect on the environment. In the previous research that has been published, academics have presented in-depth analyses of the definition of emotional intelligence (EQ) and the primary factors that contribute to its enhancement. These studies have served as an important reference for this research. The previous research does, unfortunately, suffer from several deficiencies. Even though some earlier research focused on the effect of environmental governance on EQ (such as environmental legislation, enforcement of the environment, and environmental investment), the majority of studies only examined one of these aspects. It did not combine them for a comparative health study with other socio-economic factors. This will almost certainly be detrimental to elucidating the full role that the government plays in China's system for the governance of the environment. In a similar vein, previous research has not investigated the ENVIRONMENT KUZNETS CURVE phenomenon under such governance factors. A better understanding of the link between good governance and environmental quality is less likely to emerge as incomes rise.

## Data and methods

### Theoretical background

Researchers have dedicated a significant amount of time and energy to analyzing the relationship between rising economic activity and carbon emissions (73) explained the link using the ENVIRONMENT KUZNETS CURVE model, which depicts the relationship as an inverted U-shaped curve. According to this model, when a country is in its early stages of development, its primary focus is on industrialization in order to achieve higher economic growth. However, at this stage, the country does not pay sufficient attention to environmental concerns, which leads to an increase in pollution. However, once they have reached a significant level of income, economies begin to place their primary focus on reducing carbon emissions through the implementation of cutting-edge technologies as well as stringent environmental policies and regulations (74).

In response to the growing severity of environmental pollution issues, the central government of China has clarified the environmental protection responsibilities of local governments through the enactment of laws and regulations. The exercise of environmental governance can be carried out in various ways by local governments, including the promulgation of regulations and environmental laws, the strengthening of law enforcement, and the increase in investment. The primary means by which local governments in China carry out their environmental governance responsibilities are environmental law enforcement, environmental legislation, and environmental investment. This is a conclusion that can be drawn from examining the environmental protection policies and procedures currently in place in China (75).

We are operating under the presumption that the intangible role played by temperature cannot be ignored, in addition to the direct impact that CO_2_ has on the residents' health. Climate change and an increase in temperature are both caused by carbon emissions. Heatwaves caused by high temperatures have significantly contributed to an increase in mortality, respiratory morbidity, and infectious diseases. However, the state of an individual's health is notoriously difficult to assess. In previous research, life expectancy, hospitalization rates for related diseases, and self-assessed data from micro surveys were the primary importance measures. Because it causes damage to the regulatory system, high temperatures may play a mediating role in the association between carbon emissions and joint health (76). In addition, because of the effects of global warming, pathogens and the insects that spread them are provided with good living and reproductive conditions, which speeds up the spread of infectious diseases and puts the health of humans at risk (77). As a result, high temperatures may serve as an important intermediary in the relationship between carbon emissions and the health of residents.

### Data type and sources

**Figure 1:**
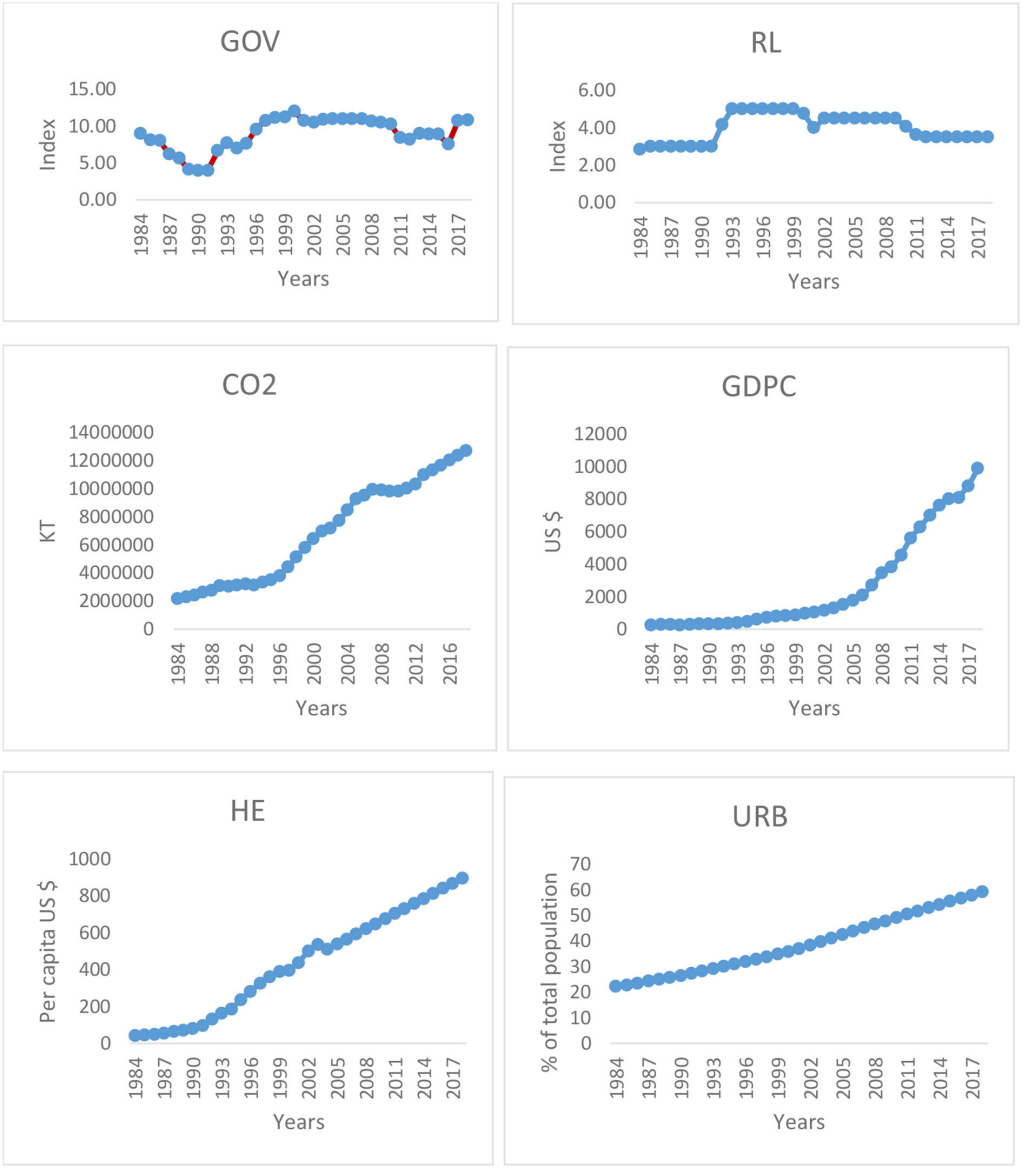
Trend graph.

For this study, annual data on the Chinese economy were collected from 1984 through 2018. The World Bank and the World Bank Institute were the primary data sources. The beginning of the study is timed to coincide with China's more recent period of economic expansion, which began in the 20th century. In addition, the sample period was chosen based on the availability of data. It is possible to consider it as a representative of the region as a whole, including the countries that originate from China. In addition, the data description can be found in Table 1, which can be found below. In addition, the trend graphs of the variables that were chosen are presented in Figure 1.

**Table 1:** Description of variables.

**Variable**	**Unit**	**Source**
CO_2_	Carbon emissions (Kt)	WDI
GDPC	Economic development (GDP US current $/Population)	WDI
URB	Urbanization (% of the total population)	WDI
HE	Health expenditures (per capita in US $)	WDI
RL	The rule of law index	ICRJ
GOV	Governance index	WGI

### Model specification

Our investigation is predicated on a well-known conceptual framework known as the Environmental Kuznets Curve, or ENVIRONMENT KUZNETS CURVE. This framework was first proposed by Kuznets (73). By reading the work of (15, 78) and adding urbanization, health expenditures, governance, and the rule of law to the KEC model, we can make the model more comprehensive. The following outlines our extended ENVIRONMENT KUZNETS CURVE model:


(1) $$\begin{array}{l} {\text{CO}}_{\text{2}}\text{=f}({\text{GDPC}}^{\text{β1}},{\text{S-GDPC}}^{\text{β2}},{\text{URB}}^{\text{β3}},{\text{HE}}^{\text{β4}},{\text{GOV}}^{\text{β5}},\\ {\text{RL}}^{\text{β6}},{\text{e}}^{\text{μ}} \end{array}$$

By taking its natural log on both sides,


(2) $$\begin{array}{l} {\text{Lco}}_{\text{2}}{\text{=β}}_{\text{0}}{\text{+β}}_{\text{1}}{\text{LGDPC+β}}_{\text{2}}{\text{LGDPC}}^{\text{2}}{\text{+β}}_{\text{3}}{\text{LURB+β}}_{\text{4}}\text{LHE}\\ \;+{\text{β}}_{\text{5}}\text{GOV + }{\text{β}}_{\text{6}}\text{RL+μ} \end{array}$$

Whereas, LCO2, LGDPC, LS-GDPC, LURB, LHE, GOV, and RL are all abbreviations for the natural log of carbon emissions, economic development and its square, urbanization, and health expenditures, respectively. In a similar fashion, the indexes of the rule of law and governance are abbreviated as GOV and RL.

### Estimation strategy

#### Priori tests

As part of the a priori analysis, the unit root test is performed so that the series integration order can be determined. We combine the Zivot unit root test with Augmented Dickey-Fuller (ADF) test given by Kao (79). An important advantage of the Zivot unit root test over traditional approaches is that it considers break dates, which are known to affect the accuracy of traditional tests. The Zivot analysis resulted in discovering the cutoff dates for each of the variables by following these steps. Cointegration evidence, as demonstrated by the co-integration test developed by Johansen (80), is also necessary in order to investigate the long-run relationship between variables in light of the a priori investigation. Because of this, additional co-integration tests using FMOLS, DOLS, and the Canonical Cointegrating Regression (CCR) techniques are being carried out as a result. After that, the ARDL method is used to look at the short- and long-term effects.

#### Cointegration test

The co-integration test compares multiple time series datasets that use a linear combination of variables to determine whether or not there are long-term relationships or whether or not there is long-run equilibrium. The Johansen co-integration test is used in this investigation to determine whether or not the relationship between variables can maintain its constancy and long-term equilibrium by using the next equation:


(3) $$\Delta {\text{Y}}_{\text{t}}\text{=}\Pi_{\text{t}-\text{1}}\text{+}\sum_{\text{i=1}}^{\text{p}-\text{1}}\Gamma \text{yt 1}{\text{+B}}_{\text{xt}}{\text{+μ}}_{\text{t}}$$

According to the equation that was just presented, it is a determinant of the adjusted disequilibrium matrix. The rate of change of endogenous factors is sped up to fight disequilibrium, which causes the stacking coefficient to be increased. When attempting to capture the short-term dynamic adjustment, the sign is utilized. This test progression has the potential to declare the association between variables and the positions they hold in the matrix, as well as featuring roots.

#### The FMOLS, DOLS, and CCR

This study utilized a fully modified ordinary least square (FMOLS) and dynamic ordinary least square (DOLS) as estimation techniques. Pedroni (81) initially developed FMOLS regression technique which is a residual-based test which provides efficient results for cointegrated variables. Moreover, FMOLS is considered as a reliable estimate when the sample size is small and eliminate the problems of endogeneity and serial correlation among the variables. Stock and Watson (82) developed a DOLS estimation in 1993. DOLS provide better results than FMOLS and eliminate correlation among regressors (83). We also employed canonical cointegrating regression (CCR) estimation as a robust estimation to validate the results of FMOLS and DOLS. Where we first employed a unit root test to check the stationarity of data used in this study. In the second step, we employed the cointegration test to check whether variables are associated in a long run or not. In the third step, we employed FMOLS (fully modified ordinary least square) and DOLS (dynamic ordinary least square test to analyze the impact of different determinants on electricity consumption. In the last step, we employed Granger Causality as a robust test to explore the causal relationships between different variables.

This study makes use of the DOLS estimator, in addition to the benefits offered by the FMOLS estimator (81). It is recommended to use the estimators rather than the OLS because the estimators use the lags and leads of first-differenced regressors, which allows them to account for a small sample bias.


(4) $${\text{X}}_{\text{t}}={\text{x}}_{\text{t}-\text{1}}+{\text{ε}}_{\text{t}}$$

Such that xt (rinvest, renp, gdp, and gdpsq) of parameter vector β for all xt such that t = 1984, 1985, …, 2018 and ε t is the error terms.

Thus, the FMOLS is expressed as follows,


(5) $$\begin{array}{l} {\text{B}}_{\text{FMOLS}}\text{=}{\left\{\sum \sum_{\text{t=1}}^{\text{T}}\left(\text{Xt}-\text{X}-\text{bar}\right)\text{(Xt}-\text{X}-\text{bar)}\right\}}^{-1}\times \\ \;\left\{\sum \sum_{\text{t=1}}^{\text{T}}\left(\text{Xt}-\text{X}-\text{bar}\right)\text{(ECEM}-\text{bar}-\text{T Δ}\in \text{μ)}\right\} \end{array}$$

For this reason, the DOLS estimator is a useful addition to co-integrating regression.


(6) $$\begin{array}{l} \text{ECE}{\text{M}}_{\text{t}}=\text{ α }+\text{ β}{\text{X}}_{\text{t}}\sum_{\text{k}=\;-\text{p}1}^{\text{p}2}\text{ƛ KΔECEM t}-\text{k}\\ \;+\sum_{\text{k}=\;-\text{q}1}^{\text{q}2}\text{ƛKΔx t}-\text{k}+\text{μt} \end{array}$$

Three vectors represent the dependent and independent variables, respectively: a co-integrating vector (), a dynamic vector (), and an intercept (). In addition, the CCR estimator is used.

#### Robustness and diagnostic tests

To confirm the precision of the estimation results, a battery of diagnostic procedures is carried out. Some previous tests demonstrates that there is statistical proof to support the hypothesis that the estimated model follows a normal distribution, free of concerns regarding serial correlation and heteroskedasticity.

#### Granger causality test

The Granger causality test estimated by the following equation:


(7) $${\text{X}}_{\text{t}}\;=\;{\text{α}}_{0}+\sum_{\text{j}=1}^{\text{k}}\text{α}1\text{sXt}-\text{s}+\sum_{\text{j}=1}^{\text{k}}\text{α}2\text{sXt}-\text{m}+{\text{ε}}_{1\text{t}}$$

(8) $${\text{Y}}_{\text{t}}=\;{\text{β}}_{0}+\sum_{\text{j}=1}^{\text{k}}\text{β}1\text{sXt}-\text{j}+\sum_{\text{j}=1}^{\text{k}}\text{β}2\text{sXt}-\text{h}+{\text{ε}}_{1\text{t}}$$

According to the above equation, it is supposed that 1t and 2t are uncorrelated. The equation shows the unidirectional causality between fatal and work accidents and four specific causes. If the estimated coefficient α2i is statistically significant, then if α2i ≠ 0, then Y Granger results in X. β2h. It is only statistically significant when the correlation coefficient between the two variables is greater than zero, i.e., β2h ≠ 0 is statistically significant. Two specific variables are linked by the significance of 2i and 2h. If α2i and aβ2h are both zero, the terms Y and X will be independent.

## Results and discussion

A descriptive investigation of the variables is presented in Table 1. The least unpredictable variables are those dealing with governance and regulations. The volatility of health expenditures is higher than that of urbanization, economic development, and emissions, but the volatility of CO2 is lower than that of FDI. In addition, there is not a sizable gap between the values of the selected variables' mean and median distributions (Table 2).

**Table 2:** Descriptive statistics.

	**LCO_2_**	**LGDPC**	**LURB**	**LHE**	**GOV**	**RL**
Mean	0.6374	0.7189	0.4132	3.9854	0.3657	0.9612
Median	0.5932	0.6547	0.3574	1.6541	0.3198	0.8823
Maximum	1.8562	1.5236	0.8956	2.9654	0.8574	1.2351
Min.	0.0023	0.6321	0.0142	0.6321	0.2314	0.8541
St. Dev.	0.3930	0.3285	0.5214	0.3041	0.1842	0.9630

Table 3's correlation matrix indicates that, at the 1% level of significance, the income per capita has a positive correlation with carbon emissions. At the same time, urbanization has a positive correlation with the variable that is being explained, and this correlation is significant at the 1% level. In addition, a positive correlation can be found between emissions and the expenditures for health care and the rule of law. On the other hand, governance has been shown to have a negative correlation with carbon emissions at a significant level of 5%.

**Table 3:** Pairwise correlation test.

	**LCO_2_**	**LGDPC**	**LURB**	**LHE**	**GOV**	**RL**
LCO_2_	1					
LGDPC	0.6321^*^	1				
LURB	0.7186**	0.7456^*^	1			
LHE	0.5687^*^	0.6523**	0.6854^*^	1		
GOV	−0.3546**	0.6921^*^	0.5632**	0.4523^*^	1	
RL	0.2341^*^	0.4521**	−0.2347**	0.6158**	0.4598^*^	

* Significance at 1%.

### Unit root test

The outcomes of the unit root test are presented in Table 4, and the unit root tests ADF and Divot are utilized in this investigation. According to the outcomes that were determined by the ADF unit root test, this test demonstrates that there is no co-integration at level for any of the variables, whereas the data becomes stationary after the first difference. In addition to that, the structural Divot unit root test is utilized in order to investigate the expected breaks in the data that is provided. Similarly, the structural break in emissions occurs at the level of 2002 and at the first difference in 2008, respectively. Similarly, the structural break for each variable can be seen in Table 4, which presents the data.

**Table 4:** Unit root tests.

**Variable**	**ADF**		**Zivot unit root**			
	**Level**	**1st difference**	**Level**	**Break**	**1st difference**	**Break**
LCO_2_	1.7423	−3.6952^*^	−2.6542**	1997	−5.5621^*^	2008
LGDPC	2.9632	−7.6521^*^	−3.8574^*^	2002	−6.9654^*^	2009
LURB	1.8541	−4.3215**	−3.8521^*^	1999	−9.5287^*^	2007
LHE	2.9854	−5.2387^*^	−4.6543^*^	2000	−7.6549^*^	2012
Gov.	1.8542	−3.8541^*^	−3.9654^*^	1998	−5.6387^*^	2006
RL	1.9632	−5.6381^*^	−4.6512^*^	2003	−7.1496^*^	2010

* Significance at 1%.

On the basis of the VAR lag order selection criteria, lag three is selected for work accidents by the Akaike information criterion (AIC), the Schwarz information standard (SC), and the Hannan–Quinn information criterion (HQ), whereas in fatal accidents, AIC and HQ selected lag three as an appropriate lag while SC selected lag 0 as an appropriate lag (Table 5).

**Table 5:** VAR lag order selection criteria.

**Lag**	**AIC**	**SC**	**HQ**
0	3.9546	2.0006	1.9654
1	1.5287	3.5694	2.0012
2	−3.8452^*^	1.8524	−0.0863
3	−2.6354	−3.6524^*^	−0.9632^*^

* Significance at 1%.

Table 6 contains the results of the Johansen co-integration test, which is used to examine whether or not there is a long-term connection between the variables. The findings are broken down into two sections: the Johansen trace test, on the one hand, and the maximum eigenvalue, on the other. According to the findings, the variables are co-integrated with a R0 value of 296.524 for trace and a critical value of 173.65 at a significance level of 1%. The maximum eigenvalue for none has a value of 121.963, and the critical value for none is 70.631; both of these values are significant at the 1 percent level, respectively. The findings of the co-integration analysis provided evidence of a long-term connection between the variables that were examined.

**Table 6:** Trace and eigen value test.

	**Trace value**	**0.05 Critical value**	**Prob**.
**Trace statistics**			
R_0_	296.524	173.654	0.000
R_1_	230.874	156.389	0.000
R_2_	170.962	96.234	0.005
R_3_	56.964	33.524	0.000
Maximum eigen value			
R_0_	121.963	70.631	0.000
R_1_	70.358	56.324	0.000
R_2_	32.6354	47.265	0.980
R_3_	14.875	28.342	0.635

### Long-run outcomes of FMOLS, DOLS and CCR estimators

In this study, an attempt is made to investigate the long-term effects on the environmental quality of factors such as income, its square, urbanization, health expenditures, governance, and environmental regulations. In order to achieve this aim, the present investigation uses a number of different econometric methods (Table 7). These methods illustrate the fascinating connections between the variables being explained and those that are being explained. As a result, the income per capita and its square are being used as the first determinant of environmental degradation in the investigation of the ENVIRONMENT KUZNETS CURVE hypothesis. The quadratic term of per capita income, also known as the gross domestic product (GDP), is introduced for understanding ENVIRONMENT KUZNETS CURVE. For MS-ECM and FMOLS, the coefficient of LGDP (1 > 0) and LGDP2 (2 0) advocated that a 1 percent rise in this factor leads to an increase of 0.163 percent in emission, while the square of the per capita income causes a decrease equivalent to 0.565 percent in an explained variable. This was the case because the coefficient of LGDP (1 > 0) and LGDP2 (2 0) were advocated. The ENVIRONMENT KUZNETS CURVE hypothesis of an inverse U-shaped relationship is supported by these findings for China's economy. According to the ENVIRONMENT KUZNETS CURVE hypothesis, the increase in ED during industrialization is caused by an increase in income per capita. To put it more specifically, industrialization necessitates the extensive consumption of various energy sources, which in turn results in ED. When a nation reaches a certain level of economic development, it enters a phase known as post-industrialization (84). During this time, the nation's emissions decrease as a result of changes in its economic structure as well as more stringent environmental protection laws and protocols (85, 86). The process of industrialization and economic growth frequently results in a significant increase in the amount of cheap energy and natural resources that developing economies consume. They build enormous infrastructures in order to stimulate economic growth, which necessitates the consumption of a wide variety of energy sources and, as a result, results in ED (87). China is a leading example of a country that has reported adequate economic growth. If China had focused on pollution during the early stages of its development, it would not have been able to achieve the level of economic growth that it has seen. The economic model of China, which is being followed by many other developing nations, relies heavily on the consumption of energy and places an intense emphasis on industrialization and investment. These countries prioritize achieving their goals of economic expansion and energy independence over protecting the environment (88). In addition, our findings are consistent with the research that is already published, such as a study that was conducted in 31 provinces in China (37), a study that was conducted in the United States of America that also supports the frequency of inverted u-shaped ENVIRONMENT KUZNETS CURVE (12), and a study that suggests the same results as Shahbaz et al. (2019), the study of Shah et al., (85), a case study that was conducted by Fu et al. (89), Shah et al. (87) and Shah et al. (85).

**Table 7:** Outcomes of FMOLS, DOLS and CCR estimators.

**Variable**	**FMOLS**		**DOLS**		**CCR**	
	**Coef**.	**Std. error**	**Coef**.	**Std. error**	**Coef**.	**Std. error**
LGDPC	3.6954^*^	1.3651	7.6397^*^	2.6378	4.6524**	0.8965
LGDPC^2^	−0.9623^*^	0.0235	−0.7852^*^	0.1289	−0.8866	0.2354
LURB	3.6985^*^	0.8521	5.0365^*^	1.2352	4.2541^*^	1.8952
LHE	−0.9632^*^	0.0236	−0.7123**	0.1145	−0.2389*	0.0562
GOV	−0.6321*	0.0234	−0.8927^*^	0.1278	−0.6114	0.1623
RL	0.8932^*^	0.1145	0.7852^*^	0.0123	0.8342^*^	0.1923

* Significance at 1%.

In light of the fact that URB was found to have a positive association with carbon emissions, it can be deduced that a one percent increase in this factor would lead to an increase in emissions of 3.698, 5.036, and 4.254% respectively in accordance with the requirements of FMOLS, DOLS, and CCR. This demonstrated a direct connection between CE and URB over a longer time period; however, unplanned URB may lead to the devastation of the environment on Earth. To put it another way, people move to urban areas in the expectation of having a better and more promising future there. However, if this procedure is not carefully planned, urban areas will not be able to accommodate the large number of people moving in from rural areas. This puts strain on a variety of urban amenities, including sanitation, and sewerage systems, which in turn contributes to the deterioration of the surrounding environment. In addition, as a result of the massive flood of people and the unplanned housing societies, there has been observed an increasing drift of deforestation, which has further contributed to the deterioration of the situation (90). There is also evidence for an inverse relationship between the CE and URB nexus in Nigeria's economy, according to Kaleem Khan et al. (91). This was found in their research on the Nigerian economy. According to Camana et al. (92)'s research, urbanization can lead to an increase in CE in the context of a developing economy like Pakistan. Therefore, it is imperative that developing economies place their attention on urbanization that is sustainable. Also this outcome is in line with findings of Shah et al. (87).

In addition, the amount spent on healthcare is increasingly being considered a proxy for the quality of the surrounding environment. Because it has a negative association with carbon emissions, we can deduce that carbon emissions are on the rise while simultaneously health care costs are falling. According to the calculated coefficients, a positive shock of one percent that will occur in the health expenditure will cause a decrease in long-term carbon emissions of 0.963, 0.712, and 0.238%, respectively. This would be one possible justification. When it comes to energy efficiency, CO_2_ emissions, and waste management, improvements are made in the transportation and equipment involved in health care systems. If people begin to view environmental protection as a luxury good, then the demand for environmental protection will increase after the demand for the common public good has been met. The level of the Environmental Kuznets Curve is associated with the indirect effect; at some level, the government invests in dipping air pollution, while at some level, the environment is compromised for the sake of reaching growth (93). The level of gross domestic product is closely related to energy consumption, which has a significant impact on the environment (94). Can et al. (95) discovered a positive association between health expenditures and CO_2_ for B&RI countries; our findings, on the other hand, show that there is no such correlation between the two variables. Appolloni et al. (96) also provided evidence that their findings vary depending on the country and level of analysis. In addition, their methodology did not incorporate a distinct measurement for the differences between private and public health expenditures. In addition, our research utilized the quantile regression method, which was not utilized by any of the earlier studies referenced in the section of the paper devoted to the literature review. As a result, this research provides fresh perspectives on the impact that public and private health care spending have had on the deterioration of the environment. In addition to the result that spending on health care in both the public and private sectors led to a reduction in the environmental pollution in Asia, This study also shows that the private health sector has a greater negative impact on CO_2_ emissions than the public sector. This result is based on comparing the two sectors' expenditures on healthcare (90).

In a similar vein, sound governance can be an important factor in preserving the environment. According to the values that have been given for the coefficient, an inverse association between governance and carbon emissions can be seen. It can be deduced from this that an increase of one percent in good governance would result in a reduction of carbon emissions of 0.632 percent (FMOLS), 0.892 percent (DOLS), and 0.611 percent (CCR), respectively. It is possible to attribute the government's attitude toward the formulation and implementation of effective policies that are effective in controlling the worsening of the environment to the fact that these policies and regulations have been implemented. There is a possibility that efficient governance can cut down on the frittering away of resources. In addition, the government plays the primary role in the correlation between the implementation of environmentally friendly policies and the quality of the environment, but it works to undermine this correlation by minimizing the purely positive effect that governance has on CO_2_ emissions (97). The established economic systems can exercise control over the illegal activities that contribute to a decline in the quality of the environment. The term “transparency” refers to how rules and regulations are adhered to in such a way that information is made openly available and can be accessed straightforwardly. As the economy expands, governments are more likely to impose appropriate regulations in order to prevent any market failures from leading to an increase in pollution and as a response to growing public awareness of the state of the environment. They can implement environmental regulations with the help of governance, which in turn helps to reduce CO_2_ emissions. As a result, decision-making processes regarding the regulation and administration of natural resource management and environmental protection are carried out effectively in these nations. The findings of the study are in line with what was expected (7). However, the findings that we obtained from this research are more solid and trustworthy than others because we utilized estimation strategies that account for heterogeneity (85).

Environmental regulations have a significant impact on environmental quality. The values presented for the rule of law suggested that a positive association exists between it and carbon emission. To put it another way, a strict rule of law tends to lower carbon emissions across the globe. A robust rule of law helps ensure the implementation of environmental policies and compels businesses to adhere to the policy guidelines and environmental protocols that have been established. The rule of law is essential for all nations, regardless of their level of income or level of development, to achieve lower carbon emissions. As a result, the rule of law in China's economy is not robust enough at this stage to adequately control the level of pollution. In other words, the estimates suggest that an increase in the number of regulations about climate change may increase the amount of carbon dioxide emissions produced by institutions that are of average quality. Such institutions may be less effective at enforcing the significant number of new regulations passed recently. A reward and penalty system should be announced by the governments of China to ensure that businesses adhere to environmental rules and regulations. Under this system, the governments should reward firms that comply with environmental regulations and penalize firms that violate environmental regulations (90).

### Model stability test

According to the given results in Table 8, this study performs the SERIAL, ARCH, WHITE and RAMSAY tests. According to the given *P*-values, there is no serial correlation in the selected model.

**Table 8:** Model stability tests.

**Test**	**F-Statistics**	***P*-value**
Serial correlation test	3.9654	0.119
ARCH test	1.8521	0.954
WHITE test	0.8956	0.741
RAMSAY test	1.2731	0.628

### Granger causality test

Although the long-run results produced by certain econometric techniques produce very interesting results, these techniques cannot elaborate on the causal effect that each variable has on its own. As a result, the Granger causality test is utilized in this investigation to estimate the causal association that exists between variables. The results are presented in the Table 9. There was a causal relationship between carbon emissions and economic development. This explains why any change in development will cause a deterioration in the quality of the environment and vice versa. The second finding was a positive bidirectional causal link operating between health care costs and carbon emissions. It indicates that increased health expenditures to finance health infrastructure may increase carbon emissions because these expenditures involve activities based on construction. On the other hand, the level of carbon emissions creates health hazards that may impact public health and may have an effect on it; this may lead to increased healthcare costs. With this in mind, Zhang et al. (98) performed an analysis to determine the carbon footprint of the health industry in Australia. They discovered that the use of renewable energy in the health industry in Australia resulted in a decrease of ~14% of the industry's overall carbon footprint. In addition, there was a causal link that went in both directions between the urban population and the amount spent on health care. It shed light on the possibility that increased urbanization might be able to support more advanced medical facilities, which might lead to an increase in health care costs.

**Table 9:** Granger causality test.

**DV**	**Type of granger causality**						
	**Short run (lag)**						**Long run**
	**ΔLCO_2_**	**ΔLGDPC**	**ΔLURB**	**ΔLHE**	**ΔGOV**	**ΔRL**	**ECT-1**
	**F-statistics [** ***P*** **-values]**						**t-stat**
ΔLCO_2_	-	3.67521 [0.000]	1.2598 [0.093]	3.24781 [0.005]	1.79524 [0.0911]	1.27694 [0.723]	−0.8963 [-2.8238]
ΔLGDPC	7.31472 [0.000]	-	3.46871 [0.000]	1.42603 [0.5611]	4.72093 [0.004]	1.97569 [0.363]	−0.13935 [-1.2612]
ΔLURB	4.1456 [0.000]	5.08139 [0.000]	-	2.93131 [0.003]	3.96547 [0.128]	0.32564 [0.355]	−0.37560 [-2.3815]
ΔLHE	4.3544 [0.000]	3.03278 [0.002]	5.52340 [0.000]	-	0.67769 [0.129]	0.18430 [0.259]	0.92819 [ 0.6178]
ΔGOV	1.9438 [0.170]	1.9732 [0.401]	7.3741 [0.000]	2.68452 [0.402]	-	5.4384 [0.000]	−0.98451 [-1.5043]
ΔRL	1.6912 [0.293]	1.6321 [0.223]	1.36974 [0.307]	2.98541 [0.098]	7.5083 [0.000]	-	−0.06279 [-1.2473]

On the other hand, increased health expenditures might suggest that the government invest in the health infrastructure. Better health facilities could encourage people to move from rural areas to urban ones, beneficial to urbanization. In their research, Shittu et al. (99) noted that the growing population in Pakistan contributed significantly to the deterioration of the environment by raising the level of carbon emissions produced by the country's transportation industry. In contrast to the findings of this study, Khan et al. (91) found, through the use of the FAIR health database, that an increased agglomeration of hospitals in urban regions brings about a reduction in the cost of laboratory testing facilities, which in turn brings about a reduction in overall health expenditures. In addition, it was discovered that there is a connection that runs in both directions between urbanization and economic development. This is because the two factors that contribute to environmental degradation are interconnected with each other. In addition, rapid urbanization has occurred worldwide as a consequence of improved access to opportunities in the areas of health care, education, and employment. In the most recent experiment, the feedback hypothesis between governance and the rule of law was successfully tested. To put it another way, the policies relating to RL and governance are cooperating. On the other hand, one-way causality existed between per capita income and governance, urbanization and emissions, health expenditures and economic development, and governance and urbanization.

## Conclusion and policy recommendations

Within the realm of economics, there is a sense of urgency regarding the repression and mitigation of the effects of climate change. For instance, the administration, legislators, and intergovernmental agencies worldwide continue to be concerned about the enormous greenhouse gas emissions produced by China. Therefore, now would be a good time to investigate whether or not an inverted U-shaped hypothesis can be validly used to determine the ecological sustainability status of the country. According to the findings of this study, the effects of economic development (GDP), the square of economic development, urbanization, health expenditures, governance, and the rule of law are mathematically important over the experimental period of 1984–2018. These findings are based on the Kuznets curve. Importantly, the findings of the study suggest that higher levels of per capita income (GDP) are associated with a deterioration in the quality of the environment during the early stages of economic development, whereas higher levels of GDP are associated with an improvement in environmental conditions during later stages of economic development. The ENVIRONMENT KUZNETS CURVE hypothesis can be considered validated for China based on this implication. Despite this, urbanization in China will inevitably lead to a worsening of the country's environmental sustainability. Despite this, governance has been shown to have a significant impact on the quality of the environment, while the rule of law has had the opposite effect. In addition, the Granger causality test is utilized in this investigation in order to determine how closely variables are linked to one another in a casual manner.

### Policy recommendations

There are a few recommendations for public policy to ensure the quality of the environment. First, if the inverted U-shaped ENVIRONMENT KUZNETS CURVE hypothesis is valid, economic growth can be viewed as a solution to environmental problems. This would follow from the validity of the hypothesis. This suggests that the growth of the country might not need to be restricted in order to control the deterioration of these environment. Instead, it needs to be supplemented with activities and technologies that improve environmental sustainability, such as reforestation projects, technologies that save energy, and other beneficial growth options to the environment. In addition, proactive policy interventions such as stringent environmental regulations ought to be enforced to ensure that the environment will be more sustainably managed while still allowing the region to experience higher growth.

The creation of technologies that reduce emissions and stringent environmental regulations are essential components of an effective strategy for lowering carbon emissions. It should also be required the government to take stringent action if businesses and consumers do not adhere to the policy protocols established to reduce CE. At the same time, these economies have a pressing need to launch new initiatives that support environmentally responsible urbanization. For instance, governments in urban areas ought to be required to plant new trees on a regular basis actively. In addition to this, urban areas need to have housing societies that have been meticulously planned. In most of the world's economies, unplanned urbanization and the growth of informal settlements wreak havoc on the natural environments. It is the responsibility of the higher authorities to improve the supervision of environmental regulation and direct humans toward engaging in friendly competition in the realm of environmental regulation. They should increase the penalties for people who tend to “free-riding” on environmental governance and promote the joint reduction of emissions through the use of financial incentives.

Therefore, if they violate their rule of law, they will suffer losses, and the length of time required to pay for health care will be significantly reduced. This will allow them to reduce the amount of money spent on health care and improve the overall quality of the health conditions of their citizens. In addition, stringent environmental regulations result in a decrease in the amount of carbon dioxide emissions, which positively influences the health of the population as a whole. Because of this, the implementation of environmentally friendly projects is of the utmost importance for developing countries. This is because access to traditional methods by health facilities is not always possible, particularly when the health facility is located far from major cities.

There are some constraints imposed by this work that is going to have to be taken into account by any subsequent works in this domain. There was a restricted amount of data spanning 1984–2018. As a result, all subsequent works will utilize the estimation method that is resistant to the aforementioned problems in addition to the other methods. Second, while the variables of governance, health expenditures, and the rule of law were all measured using the mentioned units, future researchers may use a variety of proxies to investigate the association between these factors. In conclusion, this study incorporated the variable of total health expenditures into its analysis. In the upcoming research projects, both public health expenditures and personal out-of-pocket health expenditures should be included to compare and contrast their respective effects.

## Data availability statement

The original contributions presented in the study are included in the article/supplementary files, further inquiries can be directed to the corresponding author/s.

## Author contributions

All authors listed have made a substantial, direct, and intellectual contribution to the work and approved it for publication.

## Funding

This study was supported by the National Social Science Foundation (19XMZ102) and Inner Mongolia Social Sciences Foundation (2021YB07).

## Conflict of interest

The authors declare that the research was conducted in the absence of any commercial or financial relationships that could be construed as a potential conflict of interest.

## Publisher's note

All claims expressed in this article are solely those of the authors and do not necessarily represent those of their affiliated organizations, or those of the publisher, the editors and the reviewers. Any product that may be evaluated in this article, or claim that may be made by its manufacturer, is not guaranteed or endorsed by the publisher.

## References

[1] Irfan M Shahid AL Ahmad M Iqbal W Elavarasan RM Ren S. Assessment of public intention to get vaccination against COVID-19: Evidence from a developing country. J Eval Clin Pract. (2022) 28:63–73. 10.1111/jep.13611 34427007 PMC8657341

[2] Abbas Q Nurunnabi M Alfakhri Y Khan W Hussain A Iqbal W. The role of fixed capital formation, renewable and non-renewable energy in economic growth and carbon emission: a case study of belt and road initiative project. Environ. Sci. Pollut. Res. (2020) 27, 45476–86. 10.1007/s11356-020-10413-y 32794094

[3] Huang W Saydaliev HB Iqbal W Irfan M. Measuring the impact of economic policies on Co_2_ emissions: ways to achieve green economic recovery in the post-Covid-19 era. Clim Chang Econ. (2022). 10.1142/S2010007822400103

[4] Iqbal S Bilal AR Nurunnabi M Iqbal W Alfakhri Y Iqbal N. It is time to control the worst: testing COVID-19 outbreak, energy consumption and CO2 emission. Environ Sci Pollut Res. (2021) 28:19008–20. 10.1007/s11356-020-11462-z 33184786 PMC7659900

[5] Khokhar M Zia S Islam T Sharma A Iqbal W Irshad M. Going green supply chain management during covid-19, assessing the best supplier selection criteria: A triple bottom line (tbl) approach. Probl Ekorozwoju. (2022) 17:36–51. 10.35784/pe.2022.1.04

[6] Yu X Wang P. Economic effects analysis of environmental regulation policy in the process of industrial structure upgrading: Evidence from Chinese provincial panel data. Sci Total Environ. (2021 753:142004. 10.1016/j.scitotenv.2020.142004 33207480

[7] Wu R Lin B. Environmental regulation and its influence on energy-environmental performance: Evidence on the Porter Hypothesis from China's iron and steel industry. Resour Conserv Recycl. (2022) 176:105954. 10.1016/j.resconrec.2021.105954

[8] Alola AA Alola UV Akdag S Yildirim H. The role of economic freedom and clean energy in environmental sustainability: implication for the G-20 economies. Environ Sci Pollut Res. (2022) 29:36608–15. 10.1007/s11356-022-18666-5 35066852 PMC9079036

[9] Ngo TQ. How do environmental regulations affect carbon emission and energy efficiency patterns? A provincial-level analysis of Chinese energy-intensive industries. Environ Sci Pollut Res. (2022) 29:3446–62. 10.1007/s11356-021-15843-w 34389945

[10] Hu D Jiao J Tang Y Han X Sun H. The effect of global value chain position on green technology innovation efficiency: From the perspective of environmental regulation. Ecol Indic. (2021) 121:107195. 10.1016/j.ecolind.2020.107195

[11] Luo Y Salman M Lu Z. Heterogeneous impacts of environmental regulations and foreign direct investment on green innovation across different regions in China. Sci Total Environ. (2021) 759:143744. 10.1016/j.scitotenv.2020.143744 33341514

[12] Liu W Du M Bai Y. Mechanisms of environmental regulation's impact on green technological progress—evidence from china's manufacturing sector. Sustain. (2021) 13:1–23. 10.3390/su13041600

[13] Sheng H Zhang Y Wang W Shan Z Fang Y Lyu W. High confident evaluation for smart city services. Front Environ Sci. (2022) 2022:1103. 10.3389/fenvs.2022.950055

[14] Zhang M Qiu D. Research on the impact of environmental regulations on China's regional water resources efficiency: insights from dea and fixed effects regression models. Polish J Environ Stud. (2022). 10.15244/pjoes/143846

[15] Zhang J Ouyang Y Ballesteros-Pérez P Li H Philbin SP Li Z. Understanding the impact of environmental regulations on green technology innovation efficiency in the construction industry. Sustain Cities Soc. (2021) 65:102647. 10.1016/j.scs.2020.102647

[16] Ramzan M Raza SA Usman M Sharma GD Iqbal HA. Environmental cost of non-renewable energy and economic progress: Do ICT and financial development mitigate some burden? J Clean Prod. (2022) 333:130066. 10.1016/j.jclepro.2021.130066

[17] Zhang L Huang M Li M Lu S Yuan X Li J. Experimental study on evolution of fracture network and permeability characteristics of bituminous coal under repeated mining effect. Nat Resour Res. (2022) 31:463–86. 10.1007/s11053-021-09971-w

[18] Gu W Zheng X. An empirical study on the impact of sustainable entrepreneurship: Based on the environmental Kuznets model. J Bus Res. (2021) 123:613–24. 10.1016/j.jbusres.2020.10.011

[19] Zhang Y Song Y. Environmental regulations, energy and environment efficiency of China's metal industries: A provincial panel data analysis. J Clean Prod. (2021) 280:e124437. 10.1016/j.jclepro.2020.124437

[20] Liu Y Zhu J Li EY Meng Z Song Y. Environmental regulation, green technological innovation, and eco-efficiency: The case of Yangtze river economic belt in China. Technol Forecast Soc Change. (2020) 155:e119993. 10.1016/j.techfore.2020.119993

[21] Deng Y You D Zhang Y. Research on improvement strategies for low-carbon technology innovation based on a differential game: The perspective of tax competition. Sustain Prod Consum. (2021) 26:1046–61. 10.1016/j.spc.2021.01.007

[22] Akomea-Frimpong I Adeabah D Ofosu D Tenakwah EJ. A review of studies on green finance of banks, research gaps and future directions. J Sustain Financ Invest. (2021). 10.1080/20430795.2020.1870202

[23] Chen Y kumara EK Sivakumar V. Invesitigation of finance industry on risk awareness model and digital economic growth. Ann Oper Res. (2021). 10.1007/s10479-021-04287-7

[24] Nakhli MS Shahbaz M Ben Jebli M Wang S. Nexus between economic policy uncertainty, renewable & non-renewable energy and carbon emissions: Contextual evidence in carbon neutrality dream of USA. Renew Energy. (2022) 185:75–85. 10.1016/j.renene.2021.12.04634735702

[25] Ehigiamusoe KU Lean HH Babalola SJ Poon WC. The roles of financial development and urbanization in degrading environment in Africa: Unravelling non-linear and moderating impacts. Energy Rep. (2022) 8:1665–77. 10.1016/j.egyr.2021.12.048

[26] Wu Y Zhu W. The role of CSR engagement in customer-company identification and behavioral intention during the COVID-19 pandemic. Front Psychol. (2021) 12:3171. 10.3389/fpsyg.2021.721410 34475843 PMC8407001

[27] Lei W Ozturk I Muhammad H Ullah S. On the asymmetric effects of financial deepening on renewable and non-renewable energy consumption: insights from China. Econ Res Istraz. (2021). 10.1080/1331677X.2021.2007413

[28] Quan Q Gao S Shang Y Wang B. Assessment of the sustainability of Gymnocypris eckloni habitat under river damming in the source region of the Yellow River. Sci Total Environ. (2021) 778:e14632. 10.1016/j.scitotenv.2021.146312 33725604

[29] Anser MK Yousaf Z Usman B Nassani AA Qazi Abro MM Zaman K. Management of water, energy, and food resources: Go for green policies. J Clean Prod. (2020). 10.1016/j.jclepro.2019.119662

[30] Xu X Niu D Xiao B Guo X Zhang L Wang K. Policy analysis for grid parity of wind power generation in China. Energy Policy. (2020) 138:111225. 10.1016/j.enpol.2019.111225

[31] Khokhar M Iqbal W Hou Y Abbas M Fatima A. Assessing supply chain performance from the perspective of pakistan's manufacturing industry through social sustainability. Processes. (2020) 8:1064. 10.3390/pr8091064

[32] Wang H Luo Q. Can a colonial legacy explain the pollution haven hypothesis? A city-level panel analysis. Struct Chang Econ Dyn. (2022) 60:482–95. 10.1016/j.strueco.2022.01.004

[33] Yao L Li X Zheng R Zhang Y. The impact of air pollution perception on urban settlement intentions of young talent in China. Int J Environ Res Public Heal. (2022) 19:1080. 10.3390/ijerph19031080 35162103 PMC8834384

[34] Li Z Kuo TH Siao-Yun W The The Vinh L. Role of green finance, volatility and risk in promoting the investments in Renewable Energy Resources in the post-covid-19. Resour Policy. (2022) 76:102563. 10.1016/j.resourpol.2022.102563

[35] Adedoyin FF Gumede MI Bekun FV Etokakpan MU Balsalobre-lorente D. Modelling coal rent, economic growth and CO2 emissions: Does regulatory quality matter in BRICS economies? Sci Total Environ. (2020) 710:136284. 10.1016/j.scitotenv.2019.136284 31923665

[36] Vanhercke B Ghailani D Sabato S Koch M. Social Policy in the European Union: State of Play 2018. Facing the pandemic (2021).

[37] Bashir MF Bashir MA Radulescu M Shahzad U. Investigating the role of environmental taxes and regulations for renewable energy consumption: evidence from developed economiesv. Econ Res Istraz. (2021). 10.1080/1331677X.2021.1962383

[38] Paramati SR Shahzad U Dogan B. The role of environmental technology for energy demand and energy efficiency: Evidence from OECD countries. Renew Sustain Energy Rev. (2022) 153:111735. 10.1016/j.rser.2021.111735

[39] Tang X. Innovation and Sustainability (2022).

[40] Dogan B Driha OM Balsalobre Lorente D Shahzad U. The mitigating effects of economic complexity and renewable energy on carbon emissions in developed countries. Sustain Dev. (2021) 29:1–12. 10.1002/sd.2125

[41] Yang Y Li T Wang Y Cheng H Chang SX Liang C. Negative effects of multiple global change factors on soil microbial diversity. Soil Biol Biochem. (2021) 156:108229. 10.1016/j.soilbio.2021.10822930784443

[42] Basile V Vona R. The Usefulness of Sustainable Business Models : Analysis From Oil and Gas Industry 1801–1821. (2021). 10.1002/csr.2153

[43] Dai L Wang Z Guo T Hu L Chen Y Chen C. Pollution characteristics and source analysis of microplastics in the Qiantang River in southeastern China. Chemosphere. (2022) 293:e133576. 10.1016/j.chemosphere.2022.133576 35016956

[44] Huang SZ Chien F Sadiq M. A gateway towards a sustainable environment in emerging countries: the nexus between green energy and human Capital. Econ Res IstraŽivanja. (2021) 2021:1–18. 10.1080/1331677X.2021.2012218

[45] Hermundsdottir F Aspelund A. Sustainability innovations and firm competitiveness: A review. J Clean Prod. (2021) 280:124715. 10.1016/j.jclepro.2020.124715

[46] Nasir MH Wen J Nassani AA Haffar M Igharo AE Musibau HO. Energy security and energy poverty in emerging economies: a step towards sustainable energy efficiency. Front Energy Res. (2022) 10:1–12. 10.3389/fenrg.2022.834614

[47] Salari M Javid RJ Noghanibehambari H. The nexus between CO2 emissions, energy consumption, and economic growth in the U.S. Econ Anal Policy. (2021) 69:182–94. 10.1016/j.eap.2020.12.007

[48] Wang H Hou K Zhao J Yu X Jia H Mu Y. Planning-Oriented resilience assessment and enhancement of integrated electricity-gas system considering multi-type natural disasters. Appl Energy. (2022) 315:118824. 10.1016/j.apenergy.2022.118824

[49] Jinru L Changbiao Z Ahmad B Irfan M Nazir R. How do green financing and green logistics affect the circular economy in the pandemic situation: key mediating role of sustainable production. Econ Res Istraz. (2021). 10.1080/1331677X.2021.2004437

[50] Anser MK Iqbal W Ahmad US Fatima A Chaudhry IS. Environmental efficiency and the role of energy innovation in emissions reduction. Environ Sci Pollut Res. (2020) 27:29451–63. 10.1007/s11356-020-09129-w 32445140

[51] Sulich A Sołoducho-Pelc L. Renewable energy producers' strategies in the visegrád group countries. Energies. (2021) 14:3048. 10.3390/en14113048

[52] Sun Y Li H Zhang K Kamran HW. Dynamic and casual association between green investment, clean energy and environmental sustainability using advance quantile A.R.D.L. framework. Econ Res Istraz. (2021). 10.1080/1331677X.2021.1997627

[53] Xu X Niu D Peng L Zheng S Qiu J. Hierarchical multi-objective optimal planning model of active distribution network considering distributed generation and demand-side response. Sustain Energy Technol Assessments. (2022) 53:102438. 10.1016/j.seta.2022.102438

[54] Irani F Kilic H. An assessment of implementing green HRM practices on environmental performance : the moderating role of green process. Innovation. (2022) 1:16–30. 10.5038/2771-5957.1.1.1001

[55] Gorjian S Sharon H Ebadi H Kant K Bontempo F Marco G. Recent technical advancements, economics and environmental impacts of fl oating photovoltaic solar energy conversion systems. J Clean Prod. (2021) 278:124285. 10.1016/j.jclepro.2020.124285

[56] Aboramadan M Karatepe OM. Green human resource management, perceived green organizational support and their effects on hotel employees' behavioral outcomes. Int J Contemp Hosp Manag. (2021) 33:3199–222. 10.1108/IJCHM-12-2020-1440

[57] Gupta H Kumar A Wasan P. Industry 4.0, cleaner production and circular economy: An integrative framework for evaluating ethical and sustainable business performance of manufacturing organizations. J Clean Prod. (2021) 295:126253. 10.1016/j.jclepro.2021.126253

[58] Nawaz MA Seshadri U Kumar P Aqdas R Patwary AK Riaz M. Nexus between green finance and climate change mitigation in N-11 and BRICS countries: empirical estimation through difference in differences (DID) approach. Environ Sci Pollut Res. (2021) 28:6504–19. 10.1007/s11356-020-10920-y 32997248 PMC7526081

[59] Xiao Y Zuo X Huang J Konak A Xu Y. The continuous pollution routing problem. Appl Math Comput. (2020) 387:125072. 10.1016/j.amc.2020.125072

[60] Úbeda-García M Claver-Cortés E Marco-Lajara B Zaragoza-Sáez P. Corporate social responsibility and firm performance in the hotel industry. The mediating role of green human resource management and environmental outcomes. J Bus Res. (2021) 123:57–69. 10.1016/j.jbusres.2020.09.055

[61] Yousaf Z Radulescu M Nassani AA Aldakhil AM Jianu E. Environmental management system towards environmental performance of hotel industry: does corporate social responsibility authenticity really matter? Eng. Econ. (2021) 32:484–98. 10.5755/j01.ee.32.5.28619

[62] Wang L Wang Y Sun Y Han K Chen Y. Financial inclusion and green economic efficiency : evidence from China. J Environ Plan Manag. (2022) 65:240–71. 10.1080/09640568.2021.188145935869344

[63] Wei R Ayub B Dagar V. Environmental benefits from carbon tax in the chinese carbon market: a roadmap to energy efficiency in the Post-COVID-19 Era. Front Energy Res. (2022) 10:1–11. 10.3389/fenrg.2022.832578

[64] Lin B Zhou Y. Does fiscal decentralization improve energy and environmental performance? New perspective on vertical fiscal imbalance. Appl Energy. (2021) 302:117495. 10.1016/j.apenergy.2021.117495

[65] Dudek M Spiewak R. Effects of the COVID-19 pandemic on sustainable food systems: lessons learned for public policies? The Case of Poland. Agric. (2022) 12:e10061. 10.3390/agriculture12010061

[66] Huang W Saydaliev HB Iqbal W Irfan M. Measuring the impact of economic policies on co2emissions: Ways to achieve green economic recovery in the post-covid-19 era. Clim Chang Econ. (2022) 2022:1–29. 10.1142/S.2010007822400103

[67] Fang J Kong G Yang Q. Group performance of energy piles under cyclic and variable thermal loading. J Geotech Geoenvironmental Eng. (2022) 148:04022060. 10.1061/(ASCE)GT.1943-5606.0002840

[68] Xia W Apergis N Farhan M Ghosh S. Investigating the role of globalization, and energy consumption for environmental externalities : Empirical evidence from developed and developing economies Buhari Dog. (2022) 183:84. 10.1016/j.renene.2021.10.084

[69] Huang H Chau KY Iqbal W Fatima A. Assessing the role of financing in sustainable business environment. Environ Sci Pollut Res. (2022) 29:7889–906. 10.1007/s11356-021-16118-0 34480700

[70] Alam MS Atif M Chien-Chi C Soytaş U. Does corporate R&D investment affect firm environmental performance? Evidence from G-6 countries. Energy Econ. (2019) 78:401–11. 10.1016/j.eneco.2018.11.031

[71] Iqbal W Tang YM Lijun M Chau KY Xuan W Fatima A. Energy policy paradox on environmental performance: The moderating role of renewable energy patents. J Environ Manage. (2021) 297:113230. 10.1016/j.jenvman.2021.113230 34303199

[72] Khurana S Haleem A Luthra S Mannan B. Evaluating critical factors to implement sustainable oriented innovation practices: An analysis of micro, small, and medium manufacturing enterprises. J Clean Prod. (2021) 285:125377. 10.1016/j.jclepro.2020.125377

[73] Kuznets. Economic Growth and Income Inequality on JSTOR (1955).

[74] Kinyondo A Huggins C. State-led efforts to reduce environmental impacts of artisanal and small-scale mining in Tanzania: Implications for fulfilment of the sustainable development goals. Environ Sci Policy. (2021) 120:157–64. 10.1016/j.envsci.2021.02.017

[75] Pan D Chen H. Border pollution reduction in China: The role of livestock environmental regulations. China Econ Rev. (2021) 69:e101681. 10.1016/j.chieco.2021.101681

[76] Wu H Hao Y Ren S. How do environmental regulation and environmental decentralization affect green total factor energy efficiency: Evidence from China. Energy Econ. (2020) 91:e104880. 10.1016/j.eneco.2020.104880

[77] Haldorai K Kim WG Garcia RLF. Top management green commitment and green intellectual capital as enablers of hotel environmental performance: The mediating role of green human resource management. Tour Manag. (2022) 88:104431. 10.1016/j.tourman.2021.104431

[78] Liu H Zhou R Yao P Zhang J. Assessing Chinese governance low-carbon economic peer effects in local government and under sustainable environmental regulation. Environ Sci Pollut Res. (2022) 1:1–20. 10.1007/s11356-021-17901-9 34988798 PMC8731187

[79] Kao C. Spurious regression and residual-based tests for cointegration in panel data. J. Econom. (1999). 10.1016/S0304-4076(98)00023-2

[80] Johansen S. Identifying restrictions of linear equations with applications to simultaneous equations and cointegration. J Econom. (1995) 69:111–32. 10.1016/0304-4076(94)01664-L

[81] Pedroni P. Purchasing power parity tests in cointegrated panels. Rev Econ Stat. (2001) 83:727–31. 10.1162/003465301753237803

[82] Stock JH Watson MW. A simple estimator of cointegrating vectors in higher order integrated systems. Econometrica. (1993) 61:783. 10.2307/2951763

[83] Kao C Liu ST. Fuzzy efficiency measures in data envelopment analysis. Fuzzy Sets Syst. (2000) 113:427–37. 10.1016/S0165-0114(98)00137-7

[84] Yang J Liu H Ma K Yang B Guerrero JM. An optimization strategy of price and conversion factor considering the coupling of electricity and gas based on three-stage game. IEEE Trans Autom Sci Eng. (2022). 10.1109/TASE.2022.3171446

[85] Shah MH Salem S Ahmed B Ullah I Rehman A Zeeshan M. Nexus between foreign direct investment inflow, renewable energy consumption, ambient air pollution, and human mortality: a public health perspective from non-linear ARDL approach. Front Public Heal. (2022) 9:2230. 10.3389/fpubh.2021.814208 35096757 PMC8793008

[86] Wiseman N Moebs S Mwale M Zuwarimwe J. The role of support organisations in promoting organic farming innovations and sustainability. Malaysian J Sustain Agric. (2022).

[87] Shah MH Wang N Ullah I Akbar A Khan K Bah K. Does environment quality and public spending on environment promote life expectancy in China? Evidence from a nonlinear autoregressive distributed lag approach. Int J Health Plann Manage. (2021) 36:545–60. 10.1002/hpm.3100 33351191

[88] Khosroshahi H Azad N Jabbarzadeh A Verma M. Investigating the level and quality of the information in the environmental disclosure report of a corporation considering government intervention. Int J Prod Econ. (2021) 235:108071. 10.1016/j.ijpe.2021.108071

[89] Fu FY Alharthi M Bhatti Z Sun L Rasul F Hanif I. The dynamic role of energy security, energy equity and environmental sustainability in the dilemma of emission reduction and economic growth. J Environ Manage. (2021) 280:e111828. 10.1016/j.jenvman.2020.111828 33360740

[90] Ullah I Ullah A Ali S Poulova P Akbar A Shah MH. Public health expenditures and health outcomes in Pakistan: Evidence from quantile autoregressive distributed lag model. Risk Manag Healthc Policy. (2021) 14:3893–909. 10.2147/RMHP.S316844 34584469 PMC8462281

[91] Khan SAR Ponce P Yu Z. Technological innovation and environmental taxes toward a carbon-free economy: An empirical study in the context of COP-21. J Environ Manage. (2021) 298:113418. 10.1016/j.jenvman.2021.113418 34426217

[92] Camana D Manzardo A Toniolo S Gallo F Scipioni A. Assessing environmental sustainability of local waste management policies in Italy from a circular economy perspective. An overview of existing tools. Sustain Prod Consum. (2021) 27:613–29. 10.1016/j.spc.2021.01.029

[93] Yang X Li N Mu H Ahmad M Meng X. Population aging, renewable energy budgets and environmental sustainability: Does health expenditures matter? Gondwana Res. (2022) 106:303–14. 10.1016/j.gr.2022.02.003

[94] Qudrat-Ullah H Nevo CM. The impact of renewable energy consumption and environmental sustainability on economic growth in Africa. Energy Rep. (2021) 7:3877–86. 10.1016/j.egyr.2021.05.08335805708

[95] Can M Ahmed Z Mercan M Kalugina OA. The role of trading environment-friendly goods in environmental sustainability: Does green openness matter for OECD countries? J Environ Manage. (2021) 295:113038. 10.1016/j.jenvman.2021.113038 34153584

[96] Appolloni A Chiappetta Jabbour CJ D'Adamo I Gastaldi M Settembre-Blundo D. Green recovery in the mature manufacturing industry: The role of the green-circular premium and sustainability certification in innovative efforts. Ecol Econ. (2022) 193:107311. 10.1016/j.ecolecon.2021.107311

[97] Chen YG He XLS Huang JH Wołowicz A. Impacts of heavy metals and medicinal crops on ecological systems, environmental pollution, cultivation, and production processes in China. Ecotoxicol Environ Saf. (2021) 219:112336. 10.1016/j.ecoenv.2021.112336 34044310

[98] Zhong R Ren X Akbar MW Zia Z Sroufe R. Striving towards sustainable development: how environmental degradation and energy efficiency interact with health expenditures in SAARC countries. Environ Sci Pollut Res. (2022) 1:1–18. 10.1007/s11356-022-18819-6 35171428 PMC8853387

[99] Shittu W Adedoyin FF Shah MI Musibau HO. An investigation of the nexus between natural resources, environmental performance, energy security and environmental degradation: Evidence from Asia. Resour Policy. (2021) 73:102227. 10.1016/j.resourpol.2021.102227

